# Longitudinal multiorgan transcriptomic atlas of salt-induced hypertension

**DOI:** 10.1172/jci.insight.200841

**Published:** 2026-04-23

**Authors:** Ratnakar Tiwari, Olha Kravtsova, Lashodya V. Dissanayake, Melissa Lowe, Biyang Xu, Vladislav Levchenko, Steven Didik, Ruslan Bohovyk, Daria V. Ilatovskaya, Oleg Palygin, Alexander Staruschenko

**Affiliations:** 1 Department of Molecular Pharmacology and Physiology, University of South Florida, Tampa, Florida, USA.; 2 Department of Physiology, Medical College of Georgia, Augusta University, Augusta, Georgia, USA.; 3 Department of Medicine, Division of Nephrology, Medical University of South Carolina, Charleston, South Carolina, USA.; 4 Hypertension and Kidney Research Center, University of South Florida, Tampa, Florida, USA.; 5 James A. Haley Veterans’ Hospital, Tampa, Florida, USA.

**Keywords:** Inflammation, Metabolism, Nephrology, Cardiovascular disease, Chronic kidney disease, Hypertension

## Abstract

High dietary salt intake elevates blood pressure and drives multiorgan damage. However, the molecular programs underlying progressive organ injury remain poorly defined. Here, we present a longitudinal multiorgan transcriptomic atlas of salt-induced hypertensive injury. We profiled kidney cortex, kidney medulla, heart, and liver across 4 stages, spanning early hypertension to advanced pathology in Dahl salt-sensitive rats. We identified dynamic and tissue-specific molecular trajectories, including a shared early proliferative response that converges on proinflammatory and fibrotic remodeling. Notably, we uncovered compartment-specific renal responses, showing that the cortex and medulla, despite their proximity, follow distinct molecular trajectories during disease progression. We further identified 79 stage- and tissue-specific transcription factors that drive gene expression dynamics in salt-induced hypertensive injury. Integration with human genome-wide association studies revealed conserved pathways in endocrine signaling, ion transport, lipid metabolism, and detoxification, establishing cross-species relevance and highlighting mechanistic targets of clinical importance. Compound-transcriptome analysis revealed stage- and organ-specific therapeutic opportunities, prioritizing kinase and epigenetic modulators as candidates to rebalance maladaptive gene programs. Overall, this study provides a resource for understanding molecular mechanisms from early salt-induced hypertension to tissue-specific injury and underscores the need for precision interventions.

## Abstract 2

High salt intake drives hypertension and organ damage. This study maps gene changes across organs over time, revealing shared and tissue-specific pathways and potential targets for precision therapies.

## Graphical abstract

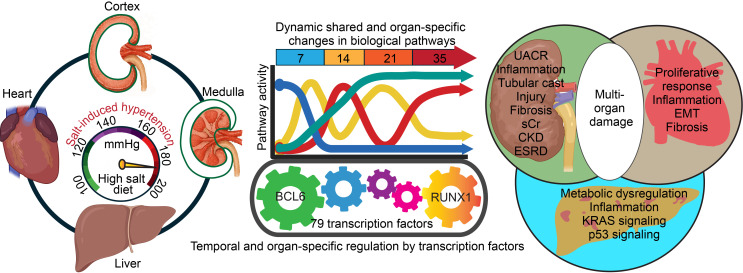

## Introduction

Hypertension is one of the most prevalent cardiovascular risk factors globally. It affects over 1 billion people and substantially contributes to stroke, heart failure, and chronic kidney disease (CKD) (1–4). Among its subtypes, salt-sensitive (SS) hypertension affects up to 30% of the global adult population and more than half of all individuals with hypertension, and it is strongly associated with CKD (5–7). Furthermore, this burden disproportionately affects high-risk groups, including African Americans, individuals with metabolic syndrome, and patients with preexisting renal disease (8). Despite its prevalence and clinical effect, salt-induced hypertension remains mechanistically complex and difficult to diagnose and manage (7, 9–11).

Although high-salt (HS) diet–induced hypertension has traditionally been studied from a renal-centric perspective, its effect extends beyond the kidney and cardiovascular system. Among other affected organs, the liver is increasingly recognized as a target of HS intake–induced metabolic reprogramming, with hepatic gene expression changes that contribute to cardiovascular disease (CVD) (12, 13). While these studies have substantially advanced our understanding of how salt-induced hypertension affects specific organs, they primarily focus on individual organs at selected time points. As a result, they fall short of capturing the full complexity of salt-induced hypertensive injury as a systemic and dynamically evolving disease. This underscores a pressing need for longitudinal, multiorgan studies that can illuminate both the cross-organ and organ-specific molecular trajectories driving disease progression over time across multiple organs.

To address this critical knowledge gap, we generated a comprehensive, temporally resolved, multiorgan transcriptomic atlas of salt-induced hypertensive injury in the Dahl SS rat model. This model remains one of the most physiologically relevant and widely utilized preclinical models of HS diet–induced hypertension. This model recapitulates key human features such as progressive hypertension, renal injury, and maladaptive cardiovascular remodeling in response to a HS diet (14–17). Previous work from our group and others leveraging different cellular, molecular, and omics approaches in this model has uncovered several important aspects of disease progression, including the activation of immune signaling pathways and metabolic reprogramming, primarily within the kidney at specific stages of salt-induced hypertension (18–22). However, the temporal dynamics of these processes across organs have not been systematically characterized.

Using advanced bioinformatics and network-based modeling, we mapped dynamic activation and suppression of molecular pathways underlying salt-induced hypertensive injury across multiple tissues. This revealed both conserved and tissue-specific molecular pathways, as well as central hub regulators that drive maladaptive responses against sustained salt-induced hypertension. Integration with genome-wide association studies (GWAS) and druggable pathway prioritization highlighted actionable therapeutic targets with potential for drug repurposing and precision intervention. Integrated with histological and biochemical analyses, this longitudinal multiorgan transcriptomic atlas delivers a systems-level view of gene expression changes in salt-induced hypertension and serves as a resource for understanding tissue- and time-specific mechanisms that contribute to chronic end-organ damage.

## Results

### A longitudinal transcriptomic atlas of salt-induced hypertension.

**Figure 1:**
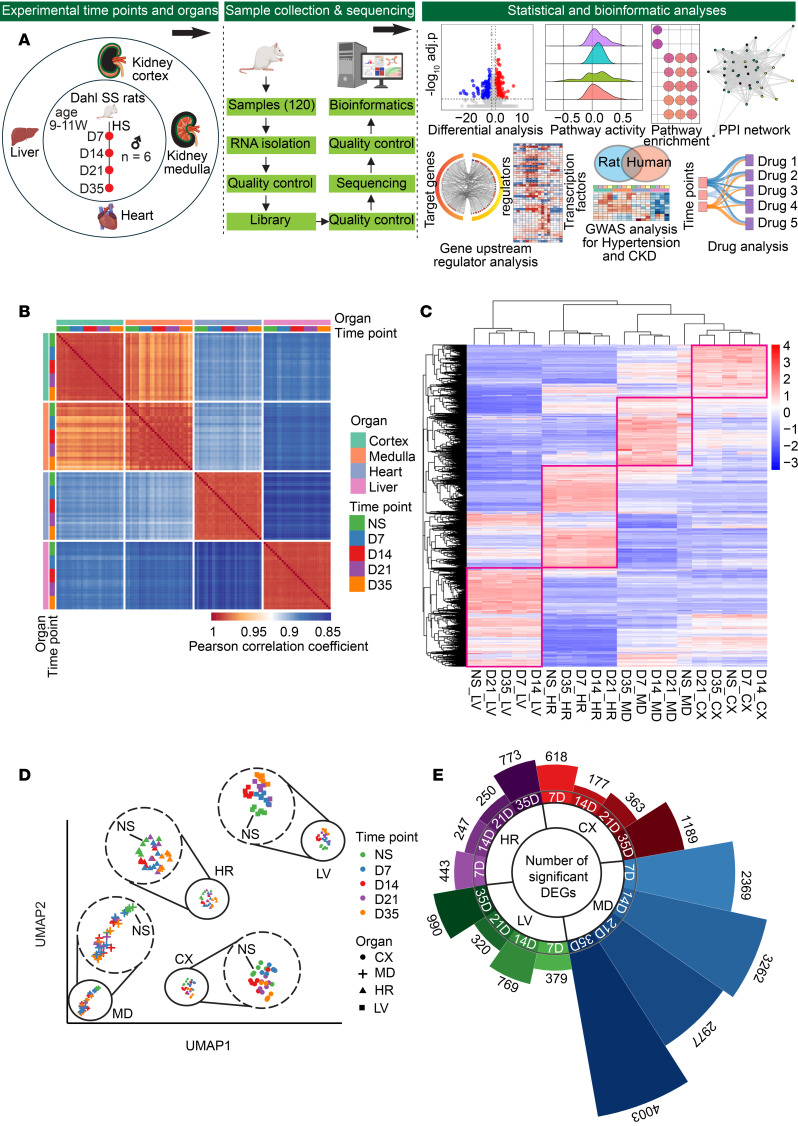
Overview of experimental design, data quality, and global transcriptional profiling. (**A**) Schematic of the study design and bioinformatics pipeline. Male Dahl SS rats were maintained on a normal-salt (NS) control diet or switched to a high-salt (HS) diet at 9–11 weeks of age to induce hypertension and organ injury. Kidney cortex, kidney medulla, liver, and heart samples were collected from NS controls and HS-fed rats after 7, 14, 21, and 35 days of treatment. RNA was isolated, quality-checked, and sequenced. Reads underwent sample-level quality control, alignment, and quantification. Downstream analyses included variance-stabilizing transformation (VST), differential expression analysis, pathway and upstream regulator enrichment, protein-protein interaction network analysis, genome-wide association study (GWAS) integration, and drug target analyses. (**B**) Sample-sample Pearson correlation heatmap, annotated by organ and time point. (**C**) Heatmap of all significantly differentially expressed genes (*P*_adj_ < 0.05), hierarchically clustered by sample (columns) and gene (rows). Red boxes highlight major coexpression gene modules segregating primarily by organ. (**D**) UMAP embedding of all 120 samples using VST counts, showing predominant clustering by organ. Dashed circles highlight zoomed-in views of distinct tissue clusters, illustrating separation of NS from HS experimental groups. (**E**) Radial bar chart summarizing the number of differentially expressed genes (|log_2_ fold change| ≥ 0.585, equivalent to a fold change ≥ 1.5, *P*_adj_ < 0.05) in each tissue and time point, illustrating dynamic and tissue-specific transcriptional responses to HS-induced hypertension. CX, cortex; MD, medulla; LV, liver; HR, heart; D7, day 7; D14, day 14; D21, day 21; and D35, day 35 time points. *n* = 6 male rats per group.

To systematically define the transcriptional landscape underlying salt-induced hypertensive injury, we performed mRNA-seq across 4 tissues: kidney cortex, kidney medulla, liver, and heart. Samples were collected from Dahl SS rats fed a HS (4% NaCl) diet at 4 progressive stages of hypertension (days 7, 14, 21, and 35), alongside a normal-salt (NS) diet–fed (0.4% NaCl) control group. This design spans the full course of disease progression, beginning with the onset of high blood pressure by day 7 (Supplemental Figure 1A; supplemental material available online with this article; https://doi.org/10.1172/jci.insight.200841DS1), through to late-stage pathology, including the terminal phase at day 35 when animals start to exhibit advanced cardiovascular dysfunction leading to stroke and death. In total, the study yields 120 high-quality transcriptomes, providing a rich resource to decode the longitudinal multiorgan molecular dynamics of HS diet–induced hypertensive injury (Figure 1A).

Principal component analysis (PCA) revealed strong segregation by organ identity (Supplemental Figure 1B), while rank-abundance plots confirm stable expression distributions across tissues and time points (Supplemental Figure 1C). Sample-sample Pearson correlation (Figure 1B and Supplemental Data Sheet 1) and hierarchical clustering of all genes (*P*_adj_ < 0.05) partitioned samples into well-defined blocks corresponding to organ (Figure 1C). In uniform manifold approximation and projection (UMAP) analysis, distinct organ-specific clusters also showed separation between control (NS) and hypertensive conditions (Figure 1D).

Next, we performed differential expression analysis, comparing the tissue samples with their corresponding NS control tissues. Applying a threshold of *P*_adj_ value less than 0.05 and absolute log_2_ fold change greater than or equal to 0.585 (corresponding to absolute fold change ≥ 1.5), we identified a substantial number of differentially expressed genes (DEGs) across the datasets (Supplemental Figure 2). We found a dynamic transcriptional pattern with an early elevation in DEGs, a midstage decline, and a subsequent rise at the later stage of HS diet–induced hypertensive injury (Figure 1E). Notably, the kidney medulla exhibited greater magnitude changes than the other tissues, with the number of DEGs increasing sharply from 2,369 on day 7 to 3,262 on day 14, decreasing to 2,977 on day 21, and peaking at 4,003 by day 35. A similar pattern was detected for the liver. However, for the kidney cortex and heart, the number of DEGs showed transient suppression of initial response earlier, on day 14, with following reactivation also peaking by day 35. These phased trajectories suggest differential responses across tissues, with the kidney medulla exhibiting the strongest transcriptional remodeling, consistent with its complex role in pressure-natriuresis and tubular transport within a relatively low-oxygen environment that increases susceptibility to injury.

To better understand the physiological context of these transcriptional changes, we next examined systemic electrolyte balance and injury (Supplemental Figures 3 and 4). The HS diet groups showed a marked increase in sodium and chloride excretion and a marked increase in blood pH, indicating a systemic change in acid-base homeostasis (Supplemental Figure 3). Furthermore, we observed an increase in relative kidney weight, a marked diuresis, and a progressive rise in urinary albumin excretion, which peaked at day 21 before partially declining by day 35 (Supplemental Figure 4). Notably, serum creatinine levels became significantly elevated only at the late stage, on day 35. Histology in the liver, heart, and kidney showed progressive injury and fibrosis, consistent with the transcriptomic signatures (Supplemental Figures 3 and 4).

Collectively, this atlas defines the dynamic transcriptomic, cellular, and functional landscape of HS diet–induced hypertensive injury.

### Salt-induced hypertension drives dynamic and tissue-specific reprogramming of core biological pathways.

**Figure 2:**
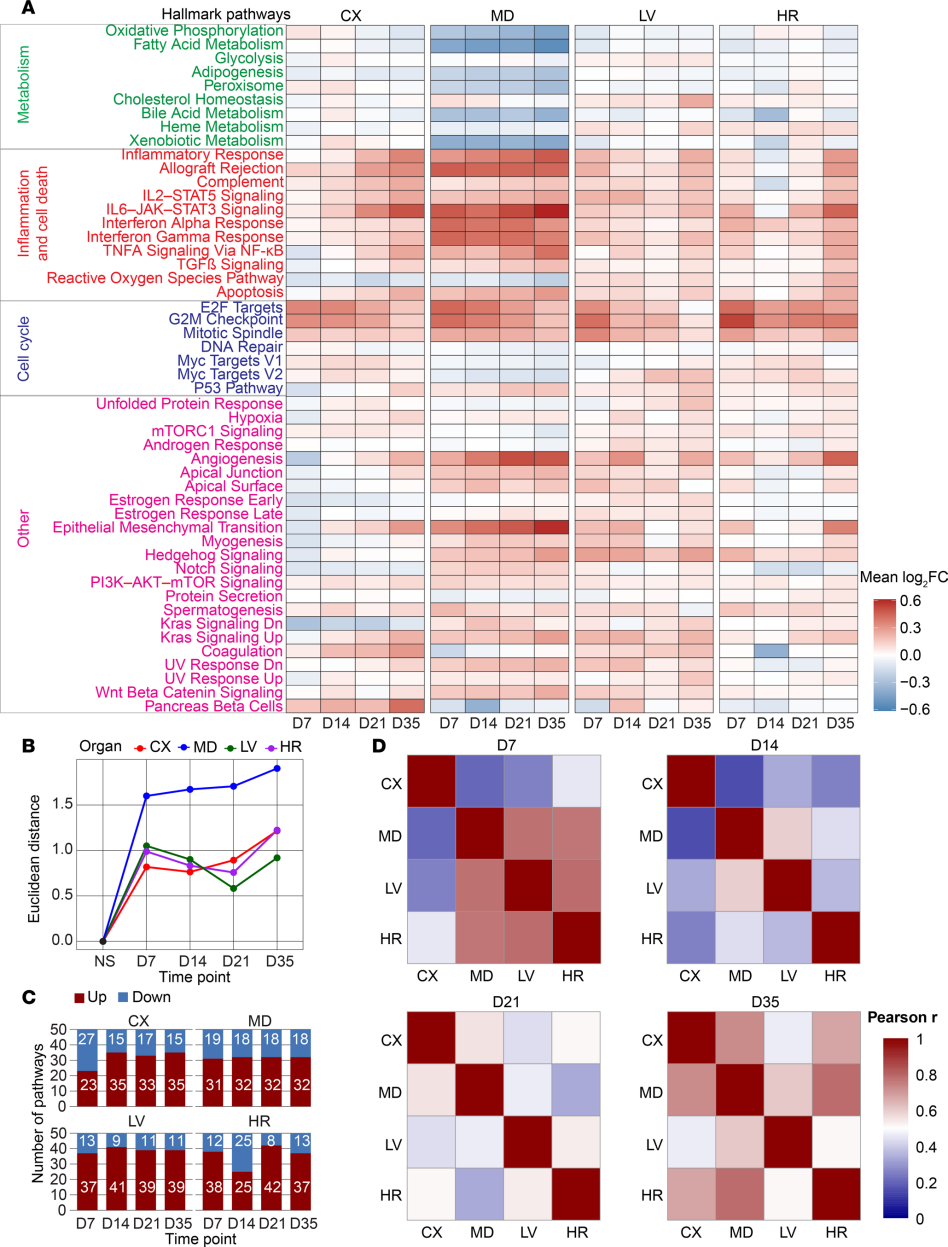
High-salt diet–induced hypertension dynamically regulates biological pathway activity across organs. (**A**) Mean pathway activity heatmap of 50 Hallmark pathways across cortex, medulla, heart, and liver at days 7, 14, 21, and 35 after initiation of a high-salt (HS) diet compared with normal-salt (NS) controls. Pathways are highlighted as functional groups, including metabolism, inflammation and death, cell cycle, and other clustered along the *y* axis. (**B**) Euclidean distances from baseline pathway profiles plotted over time, representing the magnitude of transcriptomic remodeling in each organ. The medulla shows the greatest divergence, followed by the heart, cortex, and liver. (**C**) Stacked bar charts enumerating upregulated (red) and downregulated (blue) Hallmark pathways at each time point for each organ. Numeric labels indicate pathway counts, highlighting distinct kinetics of pathway engagement across tissues. (**D**) Heatmaps showing Pearson correlation between pathway profiles across tissues (cortex, medulla, heart, and liver) at each time point (days 7, 14, 21, and 35). Each panel compares all organ pairs for a single time point, revealing similarities and differences in pathway responses among organs after the HS diet. CX, cortex; MD, medulla; LV, liver; HR, heart; D7, day 7; D14, day 14; D21, day 21; and D35, day 35 time points. *n* = 6 male rats per group.

To identify coordinated molecular responses in HS diet–induced hypertensive injury, we quantified the activity of 50 Hallmark pathways in different tissues over time. This analysis revealed coordinated activation and repression of canonical pathways with disease progression (Figure 2A and Supplemental Figure 5). As noted above, the kidney medulla exhibited the most robust and sustained changes in gene diversity and pathway activity. By day 7, inflammatory pathways were strongly upregulated and remained elevated across all subsequent time points. In parallel, proliferative and stress-adaptive programs were also activated early and sustained. In contrast, metabolic pathways were markedly suppressed at day 7 and remained inhibited as compared with the NS control medulla group. Developmental programs such as angiogenesis and epithelial-mesenchymal transition (EMT) were persistently elevated, suggesting continuous tissue remodeling responses. However, the kidney cortex showed a comparatively moderate response. Metabolic programs such as oxidative phosphorylation were slightly induced on day 7, followed by a decline over time. Inflammatory pathways were gradually upregulated, reaching peak activity at later time points. Stress pathways such as hypoxia followed a similar pattern, with late-stage elevation. Structural remodeling pathways (angiogenesis, EMT) also followed a delayed activation trajectory.

In the liver, several metabolic programs—including glycolysis, cholesterol homeostasis, and heme metabolism—together with proliferative pathways such as E2F targets and the G2M checkpoint were upregulated as early as day 7. Immune and inflammatory pathways also showed persistent activation, although at a lower magnitude than in the kidney. The heart exhibited modest activation of selected immune and stress-related pathways at day 7, followed by a transient attenuation at day 14. By day 21, a second wave of pathway activity emerged, marked by the induction of inflammation-related pathways (allograft rejection, IL-2/STAT5, IL-6/JAK/STAT3), metabolic programs (cholesterol homeostasis), and structural remodeling signals (angiogenesis and EMT).

To quantify how far each tissue’s global pathway profile deviated from its baseline state, we calculated Euclidean distances (Figure 2B). This confirmed the medulla’s early and sustained divergence, while the cortex, liver, and heart followed more gradual trajectories with moderate changes. To assess the directionality of biological pathway regulation over time, we quantified the number of upregulated and downregulated Hallmark pathways in each tissue (Figure 2C). This analysis revealed that the renal cortex exhibited a progressive shift toward increased pathway activation, with an early rise in upregulated pathways accompanied by a sustained decline in downregulated pathways. The medulla showed a consistent pattern over time, with relatively stable numbers of both up- and downregulated pathways. The liver demonstrated predominantly upregulated pathways with minimal evidence of repression, indicating sustained transcriptional activation. In contrast, the heart displayed a dynamic response, characterized by early activation, a transient reduction in upregulated pathways at the intermediate time point, and renewed activation at later stages.

To better understand whether different organs follow similar or distinct patterns of biological pathway activities during the progression of hypertensive injury, we calculated Pearson correlations using activity scores of 50 Hallmark pathways at each time point (Figure 2D and Supplemental Data Sheet 2). Notably, until day 14, the kidney medulla and cortex exhibited no significant correlation in pathway activity (D7: *r*^2^ = 0.04, *P* = 0.14; D14: *r*^2^ = 0.02, *P* = 0.28), suggesting distinct molecular responses despite their anatomical proximity. Interestingly, the medulla showed some similarity in pathway responses on day 7 with extrarenal tissues, showing significant correlations with the liver (D7: *r*^2^ = 0.60, *P* < 0.0001) and heart (*r*^2^ = 0.57, *P* < 0.0001), which dropped on day 14 (liver: *r*^2^ = 0.35, *P* < 0.0001; heart: *r*^2^ = 0.18, *P* < 0.01). On day 21, the medulla and cortex started showing similarities in pathway activity (*r*^2^ = 0.3, *P* < 0.0001), while the similarities in pathway activity between the medulla and the liver or heart dropped (Supplemental Data Sheet 2). On day 35, the similarities in pathway activities increased again, with the medulla showing significant correlations with cortex (*r*^2^ = 0.53, *P* < 0.0001), liver (*r*^2^ = 0.37, *P* < 0.0001), and heart (*r*^2^ = 0.62, *P* < 0.0001). Overall, this analysis indicates that, in the early stages of HS diet–induced hypertensive injury, tissues engage distinct biological pathway responses, with the medulla aligning more with extrarenal tissues than with the adjacent cortex. Over time, biological pathway dynamics converged across tissues, reflecting progression to a systemic state of chronic injury.

Next, we applied UMAP dimensionality reduction to the Hallmark pathway activity matrix (Supplemental Figure 6) to group pathways based on similarity in temporal dynamics, independent of absolute expression levels or tissue origin. The resulting modules captured coherent biological themes, including mitochondrial and lipid metabolism, immune and inflammatory signaling, cell cycle progression, and stress adaptation. The distinct spatial separation and tight grouping of clusters reflected coordinated engagement of different biological programs during HS diet–induced hypertensive injury, indicating synchronized activation of metabolic, inflammatory, and remodeling processes throughout disease progression.

In summary, these analyses demonstrate that HS diet induces distinct tissue-specific remodeling in the kidney, heart, and liver, while also engaging a subset of pathways that exhibit cross-organ involvement, suggesting partial convergence of molecular responses during disease progression.

### Shared molecular signatures uncover early cell cycle activation and late immune remodeling across organs.

**Figure 3:**
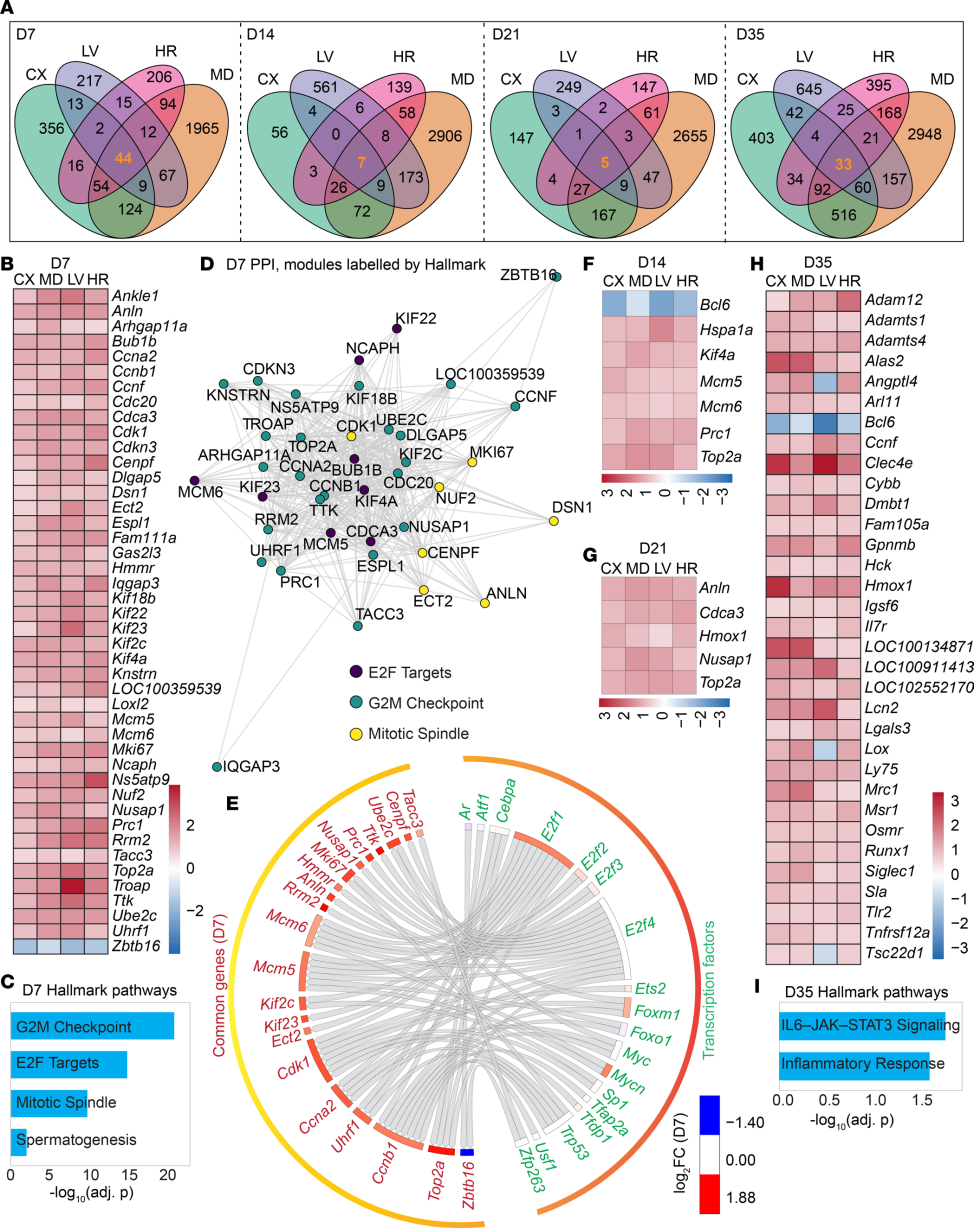
Shared transcriptional and pathway programs across organs in response to high-salt diet–induced hypertension. (**A**) Venn diagrams show overlap of DEGs across cortex, medulla, liver, and heart at each time point (days 7, 14, 21, and 35). Each segment displays the number of unique and shared DEGs. Genes shared by all tissues are highlighted in the center. (**B**) Heatmap of 44 common DEGs across all tissues on day 7. (**C**) Bar plot showing significant Hallmark pathways emerged in the analysis of day 7 common genes. (**D**) STRING-derived protein-protein interaction (PPI) network of common day 7 genes, with modules annotated by top enriched Hallmark terms per Louvain clustering. (**E**) Circos plot showing high-confidence TF–target interactions for day 7 common genes, where outer sectors represent TFs and targets, colored by average log_2_FC. Directional links denote regulatory interactions. (**F**–**H**) Heatmaps of common DEGs at days 14, 21, and 35. (**I**) Hallmark pathways emerged for D35 common genes. CX, cortex; MD, medulla; LV, liver; HR, heart; D7, day 7; D14, day 14; D21, day 21; and D35, day 35 time points. *P*_adj_ indicates *P*_adj_ value. *n* = 6 male rats per group.

To uncover conserved transcriptional programs in HS diet–induced hypertensive injury across tissues, we identified common genes that were significantly differentially expressed in all 4 tissues and may represent global targets at each time point of the disease progression. Venn analysis revealed a dynamic change of organ-specific and shared responses (Figure 3A). The early transcriptional response at day 7 showed a set of 44 genes common to all tissues. This shared signature decreased at days 14 and 21, but a distinct set of 33 overlapping genes reappeared at day 35. Heatmaps of these common DEGs demonstrated consistent expression patterns across organs within each time point (Figure 3, B and F–H). Analysis of the D7 common genes using Hallmark and Gene Ontology Biological Process (GO-BP) revealed a focused cell cycle program, including G2M checkpoint, E2F targets, and mitotic spindle terms (Figure 3C, Supplemental Figure 7A, and Supplemental Data Sheet 3). These were further supported by semantic similarity–based network analysis, which grouped top GO-BP terms into connected modules (Supplemental Figure 7B).

To examine the organizational architecture of identified global targets, we constructed protein-protein interaction (PPI) networks using STRING (23). PPI analysis on the D7 common genes formed a densely connected network, which resolved into distinct modules (Figure 3D), each enriched for canonical cell cycle processes. To further dissect regulatory architecture, we integrated high-confidence transcription factor–target (TF-target) interactions from the DoRothEA regulon (24) with day 7 common genes. This analysis revealed 18 TF genes, including forkhead box M1 (*Foxm1*), MYC proto-oncogene, bHLH TF (*Myc*), and E2F TF 3 (*E2f3*), which converged on 21 common DEGs (Figure 3E). Notably, several target genes, including cyclin A2 (*Ccna2*), DNA topoisomerase II α (*Top2a*), and cyclin-dependent kinase 1 (*Cdk1*), were coregulated by multiple TFs, underscoring a core network of transcriptional convergence tightly linked to mitotic control. In contrast, the shared gene program on day 35 displayed a stark shift in composition, with 33 common genes enriched for inflammatory and immunomodulatory mediators. The enrichment results at this time point indicated cytokine signaling IL-6/JAK/STAT3 and vascular remodeling pathways (Figure 3I and Supplemental Figure 7C). The GO-BP terms were more sparsely connected than on day 7, indicating a broader heterogeneity (Supplemental Figure 7D and Supplemental Data Sheet 3). Consistently, the day 35 PPI network did not resolve into a well-connected structure (data not shown), marking a diversified immunometabolic signature.

In summary, we identified conserved, time-dependent transcriptional programs in HS diet–induced hypertensive injury. An early proliferative response was shared across organs, whereas later stages featured a more heterogeneous immune and matrix remodeling signature.

### Organ-unique transcriptional profiles reveal the complexity of salt-induced hypertensive injury.

**Figure 4:**
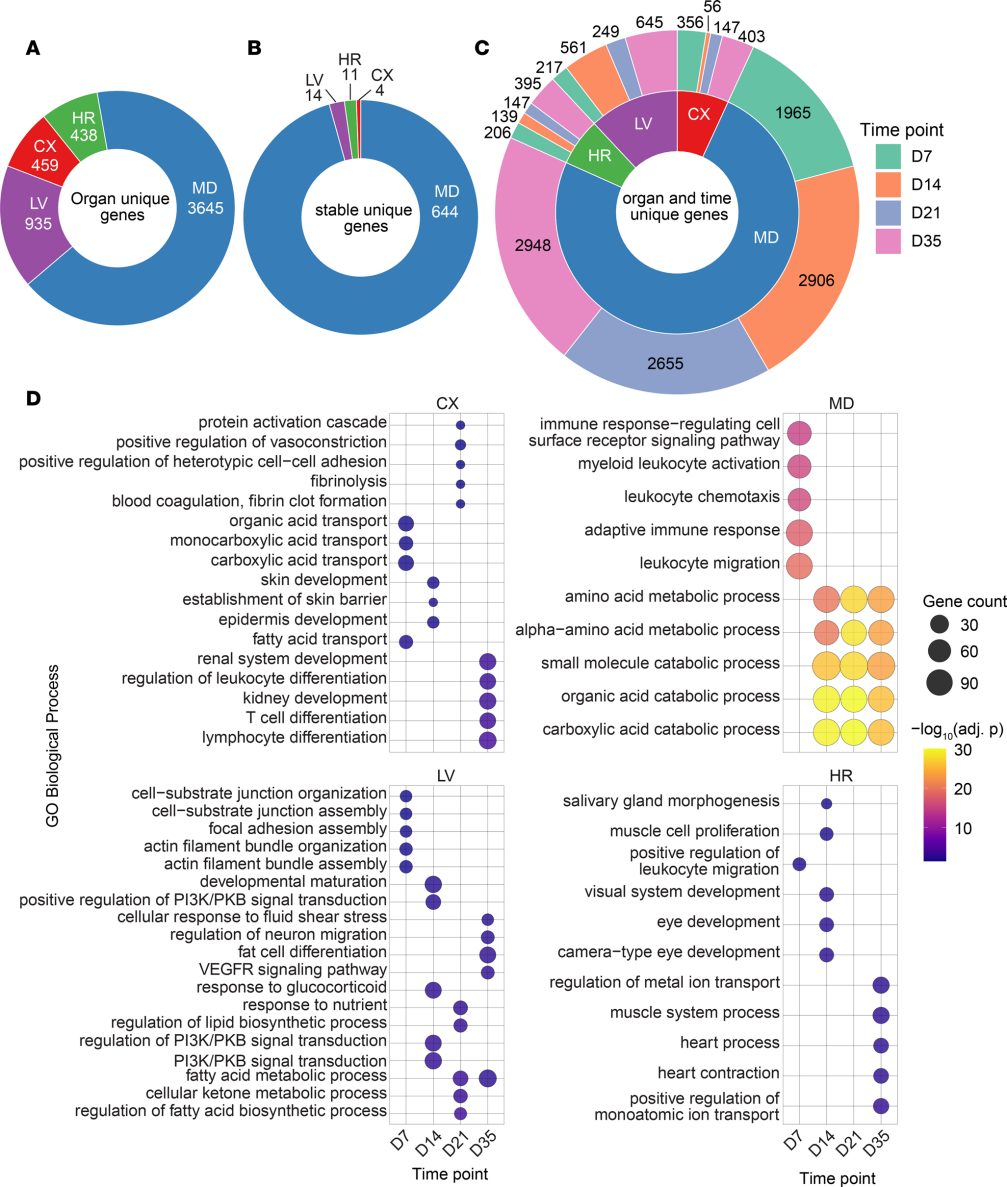
High-salt diet–induced hypertension drives distinct temporal and organ-specific transcriptional programs, revealing tissue-specific injury mechanisms. (**A**) Donut plot showing the number of DEGs that are unique to each tissue across any time point. (**B**) Subset of tissue-specific genes that are consistently differentially expressed across all 4 time points within a given tissue. (**C**) Nested donut chart illustrates the number of DEGs unique to each tissue and time point. Inner ring represents tissue, while the outer ring breaks them down by time point (days 7–35) and showing unique DEGs per tissue per time point. (**D**) Top 5 significantly enriched GO Biological Process (GO-BP) terms (*P*_adj_ < 0.05) for time point– and organ-specific DEGs. Dot size reflects the number of genes per term, and color intensity indicates statistical significance. PI3K/PKB, phosphatidylinositol 3−kinase/protein kinase B; CX, cortex; MD, medulla; LV, liver; HR, heart; D7, day 7; D14, day 14; D21, day 21; and D35, day 35 time points. *P*_adj_ indicates *P*_adj_ value. *n* = 6 male rats per group.

Beyond the global transcriptomic signatures, we next looked to more organ-specific gene remodeling. We identified organ-unique responder genes, defined as those differentially expressed in only a single tissue at any time point compared with their respective controls. This analysis revealed robust, tissue-specific transcriptomic changes (Figure 4A). The kidney medulla accounted for the largest number of organ-unique genes (3,645 genes), followed by the liver (935 genes), cortex (459 genes), and heart (438 genes), highlighting the medulla’s pronounced transcriptional responsiveness to HS diet intake (Supplemental Data Sheet 4). Next, we defined genes that remained differentially expressed across all 4 time points (day 7–35) within a single tissue (stable unique genes). This subset contained 673 genes, further dominated by the medulla (644 genes), with only limited representation in liver (14 genes), heart (11 genes), and cortex (4 genes) (Figure 4B and Supplemental Data Sheet 5). These genes represent persistent, tissue-specific responders to HS diet–induced hypertensive injury.

Next, we identified organ- and time-unique genes, defined as genes that are differentially expressed in only 1 tissue at a single time point, regardless of their expression status at other stages (Figure 4C and Supplemental Data Sheet 6). To understand the biological effect of these dynamically regulated, organ- and time-specific gene sets, we performed GO-BP enrichment (Supplemental Data Sheet 7) and plotted the 5 most significantly (*P*_adj_ < 0.05) enriched terms for each organ and time point (Figure 4D). While this focused view highlights the most prominent pathways at a specific time point, additional significantly enriched terms were detected (Supplemental Data Sheet 7). This analysis revealed that, in the kidney cortex at day 7, the top pathways were involved in organic and monocarboxylic acid transport. By day 21, top 5 GO-BP terms in the cortex shifted toward vascular and hemostatic regulation, with enrichment in pathways such as fibrinolysis, vasoconstriction, and proteolytic cascades. These features suggest the emergence of hemodynamic stress. The day 35 top 5 terms shifted toward developmental and immune differentiation, indicating late epithelial remodeling and immune engagement. Whereas in the kidney medulla, starting from day 7, enrichment was dominated by leukocyte activation and trafficking, indicating an early and robust inflammatory response. Terms related to catabolic and metabolic activities rose to the top 5 pathways at later time points in the medulla. While the immune-related pathways did not appear among the top 5 terms at later stages, they remained significantly enriched in the medulla throughout the time course of the study (Supplemental Data Sheet 7).

The liver displayed a shift from cell adhesion and focal adhesion at day 7 to lipid and fatty acid metabolic programs at days 21 and 35, including cellular ketone metabolism and fatty acid β oxidation. These terms reflect the delayed but broad metabolic reprogramming seen in the liver during HS diet–induced hypertension. The heart showed little enrichment early on, with developmental terms on day 14. By day 35, prominent enrichment for contractile and ion transport programs emerged, including heart contraction, muscle system process, and metal ion transport, highlighting structural and functional remodeling in late-stage disease.

We next extended our analysis to all DEGs per organ and time point to capture broader patterns of biological pathways in HS diet–induced hypertensive injury. We performed GO-BP (Supplemental Figure 8 and Supplemental Data Sheet 8) and Hallmark pathway enrichment analyses (Supplemental Figure 9 and Supplemental Data Sheet 9) on the complete sets of DEGs, stratified by organ and time point. Together, these analyses reveal how HS diet–induced hypertensive injury drives both temporal and organ-specific transcriptional responses, as well as broader biological processes that are active across multiple stages and shared among organs. We show that individual organs deploy distinct molecular programs while also participating in common stress-related pathways. This integrated view helps explain how different tissues respond in parallel yet uniquely to HS diet–induced hypertensive injury.

### Dynamic TF-target gene networks coordinate specific responses in salt-induced hypertensive injury.

To infer upstream regulators of transcriptomic changes, we used the ChEA 2022 database, a high-confidence resource of TF-target interactions from ChIP-seq and ChIP-chip studies (25, 26). Performing enrichment analysis of DEGs against the ChEA database identified candidate TFs likely driving the tissue- and time-specific transcriptional programs in HS diet–induced hypertensive injury (Supplemental Figure 10 and Supplemental Data Sheet 10). All TFs emerging from this analysis with *P*_adj_ < 0.05 across all tissue and time point combinations were aggregated. From this comprehensive set, we further selected TFs that were, themselves, significantly differentially expressed, thereby prioritizing TFs both statistically enriched for targeting DEGs and directly responsive to HS diet–induced hypertensive injury. This 2-step filtering yielded a final set of 79 high-confidence TFs (Supplemental Figure 11A). To investigate how TF regulation evolves across tissues and over time, we applied PCA and UMAP to expression profiles of 79 high-confidence TFs, with PCA separating tissues by major transcriptional variance and UMAP resolving tissue-specific and temporal shifts in HS diet–induced hypertensive injury (Supplemental Figure 11B).

**Figure 5:**
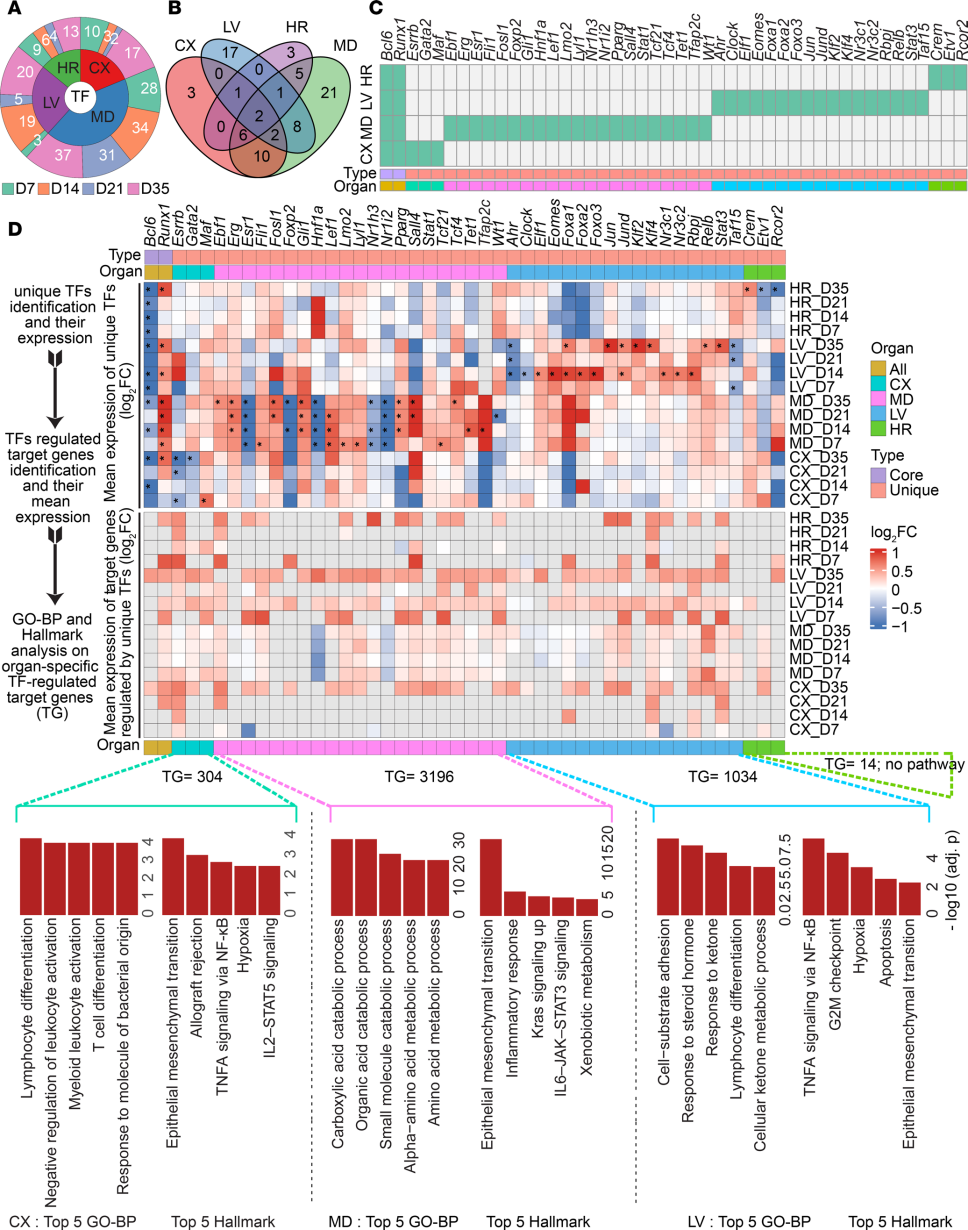
High-salt diet–induced hypertension drives organ-specific regulatory programs. (**A**) Concentric donut plot displaying the number of significantly differentially expressed transcription factors (TFs) per tissue (inner ring) and their distribution across 4 time points (days 7, 14, 21, and 35). (**B**) Venn diagram depicting the overlap and exclusivity of TFs across cortex, medulla, liver, and heart, identifying shared versus tissue-specific TFs. (**C**) Unique TFs per tissue and shared TFs visualized as a binary heatmap. (**D**) TF-target genes network and functional relevance analysis. Top heatmap: showing the expression dynamics of 2 core and unique TFs across 16 tissue–time point combinations. Asterisks denote significantly altered TFs (|log_2_ fold change| ≥ 0.585, equivalent to a fold change ≥ 1.5 and *P*_adj_ < 0.05). Middle heatmap: showing mean log_2_FC of TF-regulated target genes. Lower pathway panel: showing top 5 GO Biological Processes (left) and Hallmark pathways (right) for CX, MD, and LV emerged by TF-regulated target genes. Bar heights represent –log_10_ *P*_adj_ values. Target gene (TG) counts indicate the number of organ-specific genes regulated by corresponding TFs and used in the pathway analysis. No pathways met significance thresholds in HR. Enriched pathways with *P*_adj_ < 0.05 were considered significant. CX, cortex; MD, medulla; LV, liver; HR, heart; D7, day 7; D14, day 14; D21, day 21; and D35, day 35 time points. *P*_adj_ indicates *P*_adj_ value. *n* = 6 male rats per group.

To identify which TFs differentiate tissue-specific responses over time, we applied random forest classifiers at each time point using the expression profiles of 79 TFs as input features and tissue identity as the classification target. Feature importance was assessed using the mean decrease in Gini impurity, a measure of how much each TF contributes to accurately distinguishing tissue-specific profiles. Through this supervised classification approach, we identified TFs most responsible for separating tissue-specific responses at different phases of HS diet–induced hypertensive injury (Supplemental Figure 11C). Next, to map the temporal involvement of TFs, we identified TFs that were significantly differentially expressed in 1 tissue at a given time point. These were classified as organ-time unique TFs capturing tissue and time-restricted activity (Figure 5A and Supplemental Data Sheet 11). This analysis revealed tissue- and time-dependent heterogeneity in TF expression.

To assess the degree of overlap in transcriptional regulation across tissues, we constructed a 4-way Venn diagram of all TFs (Figure 5B). This analysis revealed 44 TF genes exhibiting organ-specific regulation. Only 2 TF genes, BCL6 transcription repressor (*Bcl6*) and RUNX family TF 1 (*Runx1*), were shared across all 4 tissues, indicating that TF activity in HS diet–induced hypertensive injury is largely tissue specific. To explore this organ specificity in greater detail and extend the overlap patterns seen in the Venn diagrams, we generated a binary presence-absence heatmap of organ-unique TFs (Figure 5C), providing a more granular view by displaying each TF individually and revealing clear segregation of TFs by tissues. Next, to assess whether organ-unique TFs impose functional control on downstream genes, we mapped ChEA predicted targets for each of the 44 TFs that remained restricted to a single tissue across the experiment. For every tissue, we compiled only the targets linked to its own unique TFs and overlaid TFs and target gene expression onto our RNA-seq data. (The expression dynamics of multiple organ-unique TFs paralleled those of their predicted targets, indicating the functional coherence of these regulatory relationships (Figure 5D). This coordinated activity suggests that TFs do not operate in isolation but, instead, regulate structured transcriptional modules that drive specific responses in HS diet–induced hypertensive injury. The magnitude of these regulons varied substantially across tissues, with medulla-unique TFs regulating the largest gene networks, followed by those in the liver, cortex, and heart. Pathway analysis of the organ-specific target pools revealed distinct biological themes. Cortex regulons were enriched for immune cell differentiation and activation, EMT, TNFA signaling via NF-κB, hypoxia, and IL-2–STAT5 signaling. Alongside, medulla regulons were dominated by catabolic and metabolic processes, Kras signaling, IL-6/JAK/STAT3 signaling, and xenobiotic metabolism. Whereas liver-restricted TFs were associated with pathways involving cell-substrate adhesion, steroid hormone response, ketone metabolism, lymphocyte differentiation, TNFA signaling via NF-κB, hypoxia, apoptosis, and EMT (Figure 5D and Supplemental Data Sheet 12).

In summary, identified 79 high-confidence TFs and mapped their dynamic, tissue-specific regulatory networks in HS diet–induced hypertensive injury, yielding a curated list of candidate targets for mechanistic and translational studies.

### Integration with the human GWAS reveals a conserved hypertension- and CKD-linked transcriptional response.

To determine whether the transcriptional programs altered in our hypertensive rat model are relevant to human disease, we integrated our transcriptomic data with human GWAS loci for the terms “hypertension” and “chronic kidney disease.” Among the 5,213 rat DEGs mapped to human orthologs, 100 genes overlapped with hypertension-associated GWAS genes, comprising a total of 386 genes (Supplemental Figure 12A), and 143 genes intersected with CKD-associated genes spanning 406 total genes (Supplemental Figure 12B). These overlaps represented highly significant enrichments (Fisher’s exact test, *P* < 0.0001; odds ratios = 2.4 for hypertension and 3.8 for CKD), highlighting that the transcriptional shifts seen in our rat model overlap strongly with known human hypertension and CKD risk loci-associated genes. Expression analysis of these overlapping genes revealed distinct temporal and tissue-specific expression patterns (Supplemental Figure 13). Further analysis across GO-BP, KEGG, and Reactome databases shows that hypertension-linked genes are dominated by developmental and endocrine pathways (Supplemental Figure 12C and Supplemental Figure 14, A–C). In contrast, CKD-linked genes were enriched for metabolic and detoxification pathways (Supplemental Figure 12D and Supplemental Figure 14, A, D, and E).

To translate the GWAS-overlap lists into mechanistic circuitry, we constructed PPI networks using STRING (confidence > 0.4 for hypertension and > 0.7 for CKD) and applied Louvain clustering (27) to identify discrete communities. With this approach, 100 GWAS overlap gene-associated proteins of hypertension were segregated into 7 modules (Supplemental Figure 12E). A connected ion-flux core (M1, cardiac muscle cell action potential involved in contraction) was anchored by calcium-handling hubs calcium voltage-gated channel subunit α 1 D (CACNA1D) and potassium inwardly rectifying channel subfamily J member 2 (KCNJ2), together with the gap junction protein α 1 (GJA1), defining an electrically responsive scaffold. A collagen-activated tyrosine kinase receptor module (M4), built around collagen type IV α 1 chain (COL4A1) and collagen type VI α 3 chain (COL6A3), linked extracellular matrix remodeling to this ion-flux hub, consistent with pressure-induced structural adaptation. The hormone-metabolic module (M3) expanded this circuitry by incorporating one-carbon and lipid transport elements, such as methylenetetrahydrofolate reductase (MTHFR), which aligns folate-dependent methyl flux with the cholesterol carrier apolipoprotein E (APOE) and a suite of steroid-modifying enzymes. MTHFR also maintained a high-confidence connection to cytochrome P450 family 17 subfamily A member 1 (CYP17A1), the anchor of the steroid-biosynthetic module (M6). This bridge places methyl-group supply in close proximity to cortisol and androgen synthesis, suggesting that folate status could influence endocrine output during the hypertensive response. In contrast, the cAMP-signaling triad (M7), containing phosphodiesterase 3A (PDE3A), phosphodiesterase 1A (PDE1A), and ectonucleotide pyrophosphatase/phosphodiesterase 3 (ENPP3), was topologically isolated, indicating that cyclic-nucleotide regulation operates as an independent signaling node.

Analysis of the CKD GWAS-overlap interactome (Supplemental Figure 12F) resolved 8 discrete communities. The M1 module, annotated as organic-acid catabolic process, represents a β-oxidation axis anchored by acyl-CoA dehydrogenase long chain (ACADL), acyl-CoA dehydrogenase medium chain (ACADM), acyl-CoA dehydrogenase short chain (ACADS), and the peroxisomal oxidase acyl-CoA oxidase 1 (ACOX1). Attached to M1, the M2 module (olefinic-compound metabolic process) includes the long-chain desaturases fatty acid desaturase 1 (FADS1), fatty acid desaturase 2 (FADS2), and the elongase ELOVL fatty acid elongase 2 (ELOVL2), showing that unsaturated fatty acid synthesis feeds into downstream β-oxidation. A multifunctional detoxification cluster emerged alongside these lipid modules, enriched for cytochrome P450 enzymes, the eicosanoid synthase arachidonate 15-lipoxygenase (ALOX15), and glutathione-handling proteins glutathione S-transferase mu 2 (GSTM2), and γ-glutamyltransferase 1 (GGT1). Edges connecting adjacent transporter-enriched communities — comprising SLC family members solute carrier family 22 member 1 (SLC22A1), solute carrier family 22 member 2 (SLC22A2), solute carrier family 2 member 9 (SLC2A9), solute carrier family 28 member 2 (SLC28A2), and the ABC transporter ATP binding cassette subfamily G member 2 (ABCG2) — suggest potential coordination in handling lipid-derived metabolites and purine catabolites through parallel excretory pathways. In parallel, several metabolic programs remained topologically insulated. A compact kynurenine module composed of kynureninase (KYNU), kynurenine 3-monooxygenase (KMO), kynurenine aminotransferase 3 (KYAT3), and arylformamidase (AFMID) forms a dedicated tryptophan-metabolism circuit. Likewise, the nucleotide metabolism group, including flap structure-specific endonuclease 1 (FEN1), thymidylate synthase (TYMS), and myelin regulatory factor (MYRF), and the developmental cluster, comprising roundabout guidance receptor 1 (ROBO1), slit guidance ligand 2 (SLIT2), and adhesion G protein-coupled receptor L3 (ADGRL3), occupy peripheral positions.

In summary, our data detect genes linked to human GWAS loci for hypertension and CKD. These conserved signatures underscore the translational relevance of our findings for dissecting multiorgan disease mechanisms in HS diet–induced hypertensive injury.

### Compound-transcriptome mapping reveals stage- and organ-specific therapeutic opportunities.

**Figure 6:**
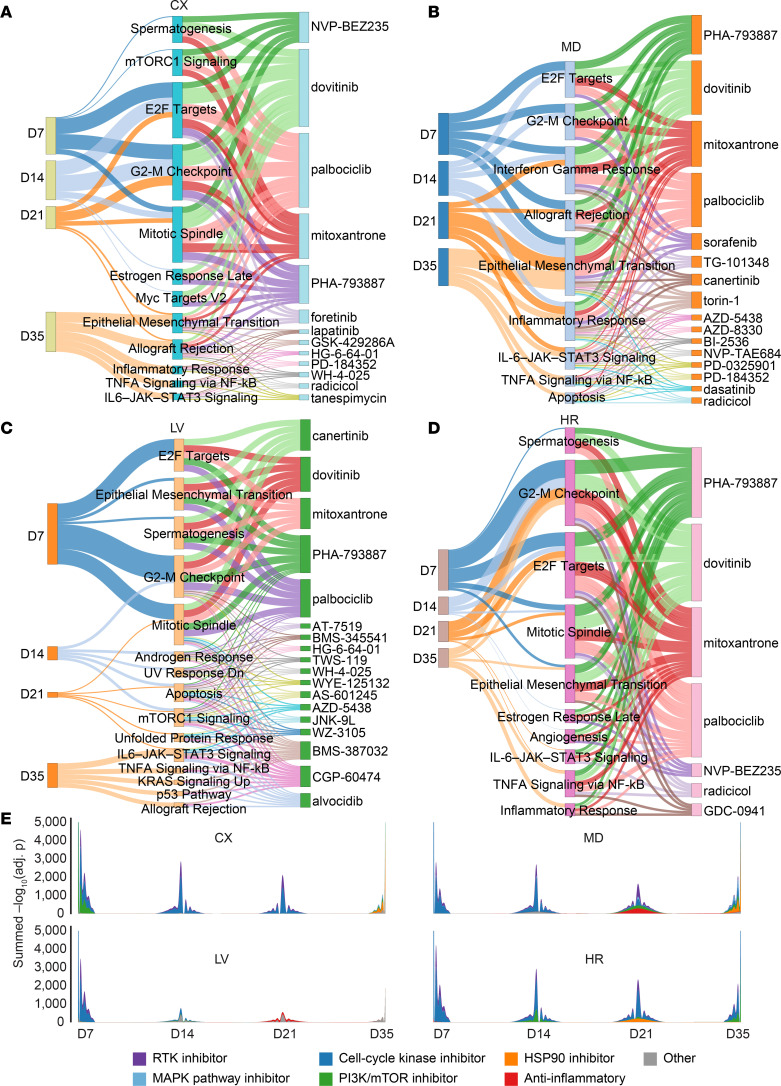
Temporal and organ-specific prediction of small-molecule modulators to target salt-induced hypertensive gene signatures. (**A**–**D**) Sankey diagrams integrating differential expression profiles with LINCS L1000 chemical perturbation signatures for significantly upregulated genes in the kidney cortex (**A**), kidney medulla (**B**), liver (**C**), and heart (**D**). Each network connects time points (days 7–35; left), enriched top 5 Hallmark pathways (middle), and the top predicted small molecules (right) prioritized for their potential to reverse disease-associated transcriptional changes. Edge thickness denotes −log_10_(*P*_adj_ value), indicating enrichment strength. (**E**) Stacked stream graphs show, for each tissue, the cumulative enrichment of small-molecule classes at each time point, represented as the summed –log_10_(*P*_adj_ value) of all compounds within each class. Peaks indicate phases when a given class is most strongly aligned with the organ-specific transcriptome. CX, cortex; MD, medulla; LV, liver; HR, heart; D7, day 7; D14, day 14; D21, day 21; and D35, day 35 time points. *P*_adj_ indicates *P*_adj_ value. *n* = 6 male rats per group.

To systematically identify compounds that could reverse HS diet–induced hypertensive injury-associated transcriptional programs, we leveraged the Library of Integrated Network-Based Cellular Signatures (LINCS) database (28, 29). Our LINCS-based predictive compound–transcriptome analysis identified several high-confidence compounds with the potential to reverse maladaptive gene profiles across organs (Figure 6 and Supplemental Figure 15). In the kidney cortex, NVP-BEZ235 (dual phosphoinositide 3-kinase/mechanistic target of rapamycin [PI3K/mTOR] inhibitor), dovitinib (fibroblast growth factor receptor/vascular endothelial growth factor receptor [FGFR/VEGFR] inhibitor), and palbociclib (cyclin-dependent kinase 4/6 [CDK4/6] inhibitor) were predicted to most effectively counter sustained induction of E2F target genes, G2M checkpoint components, and mechanistic target of rapamycin complex 1 (mTORC1) signaling pathways driving proliferative expansion and metabolic alterations (Figure 6A). These findings suggest that coordinated inhibition of growth factor receptors and cell-cycle regulators may restore transcriptional homeostasis in cortical tissue.

In the kidney medulla, PHA-793887 (multi-CDK inhibitor), dovitinib, mitoxantrone, palbociclib, and sorafenib (multi-kinase inhibitor) emerged as prominent candidates predicted to reverse maladaptive proliferative programs and concurrent activation of IFN-γ and Janus kinase/signal transducer and activator of transcription 3 (JAK/STAT3) inflammatory pathways (Figure 6B). Notably, selective mitogen-activated protein kinase (MEK) inhibitors, including PD-0325901 and PD-184352, were also associated with reversal of downregulated metabolic pathways such as fatty acid metabolism and oxidative phosphorylation (Supplemental Figure 15B), suggesting their potential to reactivate suppressed metabolism. This combinatorial signature highlights a dual proliferative-inflammatory axis as a therapeutic target in medullary injury.

In the liver, compounds such as canertinib (epidermal growth factor receptor/human epidermal growth factor receptor 2 [EGFR/HER2] inhibitor), dovitinib, mitoxantrone, PHA-793887, palbociclib, and CGP-60474 (broad-spectrum CDK inhibitor) were consistently prioritized to counter maladaptive proliferation, unfolded protein response, and NF-κB–mediated inflammatory signaling (Figure 6C). Additionally, MEK inhibitors (PD-0325901, AZD-8330, and selumetinib), along with decitabine (a DNA methyltransferase inhibitor), and withaferin A (a natural steroidal lactone), were predicted to restore downregulated transcriptional programs (Supplemental Figure 15C). These findings underscore the dual potential of kinase and epigenetic modulators to suppress pathogenic activation while reactivating metabolic pathways central to hepatic remodeling.

In the heart, enrichment was observed for PHA-793887, dovitinib, mitoxantrone, NVP-BEZ235, radicicol (heat shock protein 90 [HSP90] inhibitor), and GDC-0941 (PI3K inhibitor) to counter early mitotic gene induction and subsequent angiogenic and inflammatory activation (Figure 6D). MEK inhibitors (AZD-8330, trametinib), EGFR/ERBB family inhibitors (neratinib, erlotinib, afatinib), and radicicol were recurrently associated with restoration of downregulated programs spanning xenobiotic metabolism, adipogenesis, and estrogen response (Supplemental Figure 15D), highlighting the capacity of pathway-selective inhibitors to normalize both hyperactivated and suppressed networks.

To further resolve how pharmacologic predictions change over the course of disease, we classified compounds into mechanistic classes and quantified their enrichment dynamics across disease progression (Figure 6E). Temporal analysis revealed early enrichment of cell-cycle and receptor tyrosine kinase (RTK) inhibitors, shifting toward PI3K/mTOR and HSP90 inhibitors at later stages. These patterns demonstrate that different classes of compounds are preferentially predicted at distinct disease phases, further reflecting evolving pathophysiological mechanisms as salt-induced hypertensive injury progresses. Collectively, this integrated analysis predicts pharmacologic candidates and their effect on different biological pathways. These findings suggest the need for precision medicine approaches. Such approaches should not focus solely on lowering blood pressure but also account for the duration of hypertension, organ damage, and associated molecular pathways.

## Discussion

In this study, we demonstrate that HS diet–induced hypertensive injury progresses through a conserved early proliferative program, followed by divergent immune and fibrotic remodeling across organs. The kidney medulla exhibits the most pronounced and sustained changes, marked by robust inflammatory activation and metabolic suppression, whereas the cortex, liver, and heart show delayed, tissue-specific programs. We identified 79 high-confidence regulators, largely with organ-restricted activity, with BCL6 and RUNX1 emerging as the major conserved regulators across tissues. Integration with human GWAS loci further supplements the translational relevance of our findings, linking experimental salt-induced hypertension signatures to established hypertension- and CKD-associated genes and pathways. Compound-transcriptome mapping suggested the need for stage- and organ-specific therapeutic approaches in HS diet–induced hypertensive injury. Inhibitors of PI3K/mTOR, CDKs, and RTKs emerged as candidates for early disease stages, whereas later stages could be better ameliorated by modulators of MAPK signaling, HSP90 inhibitors, and agents targeting epigenetic regulation.

The renal medulla has long been recognized as particularly vulnerable to injury because of low oxygen gradients, high sodium transport activity, and relative hypoperfusion (10, 30). Hypoxia and oxidative stress have been implicated as key drivers of injury in this region, yet the molecular pathways linking these physiological stressors to progressive damage remain poorly defined. Our longitudinal transcriptomic analysis helps close this gap. Our data indicate that medullary injury in HS diet–induced hypertension is manifested by the early and sustained activation of immune and proliferative programs, with metabolic suppression. Together, these medullary changes may be the key drivers for progressive kidney injury and should be investigated in more detail. Notably, because albuminuria emerges as one of the earliest detectable parameters in HS diet–induced hypertension in Dahl SS rats, cortical injury is often considered central to disease progression. In earlier work, we demonstrated that cortical glomeruli and tubules undergo metabolic dysregulation, oxidative stress, and structural remodeling during HS diet–induced hypertensive injury (20). Nevertheless, it remained unresolved whether cortical alterations or the medullary injury are the major events in the HS diet–induced kidney damage. Here, we provide a direct comparison between the cortex and medulla within the kidney. We found that, despite their proximity, the cortex showed a delayed and transient transcriptional program, with a brief rise in metabolic activity followed by later immune activation. This pattern contrasted with the medulla, which showed early cast formation, cellular injury, and fibrosis, while cortical injury remained comparatively moderate. Functionally, albuminuria rose early and later slightly declined, reflecting an initial phase of hyperpermeability or hyperfiltration followed by nephron loss. Serum creatinine also increased only at a late stage, aligning with the cortical damage observed in later stages in histological analysis. Together, our study provides important clarity that the cortex initially shows a metabolically adaptive phenotype characterized by increased oxidative metabolism and glomerular hyperfiltration. Over time, however, this adaptive state shifts toward immune activation and extracellular matrix remodeling, with the medulla emerging as the primary site of injury and possibly a major driver of CKD in salt-induced hypertensive injury. Our study also underscores the limited sensitivity of conventional serum creatinine measurements for detecting early hypertension-induced kidney injury. This highlights the need for more comprehensive biomarkers capable of capturing both alterations in filtration and early medullary stress before irreversible damage occurs.

Beyond the kidney, emerging evidence suggests that salt-induced hypertensive injury also engages the liver, positioning it as an important yet underappreciated contributor to disease progression. The liver is increasingly recognized as an active modulator of systemic hypertension, metabolic dysfunction, and CVD (31, 32). Notably, epidemiological data indicate that HS intake is linked to a greater risk of developing nonalcoholic fatty liver disease and advanced liver fibrosis (33–36). Furthermore, experimental studies on murine models indicate that a HS diet induces epigenetic modifications in the liver that sustain hepatic steatosis and inflammation, ultimately contributing to cardiovascular injury (13). Within this evolving concept, our analysis offers a longitudinal perspective of hepatic transcriptional remodeling in HS diet–induced hypertensive injury. Our findings indicate that the liver actively responds to HS diet through metabolic and inflammatory signals, which may contribute to vascular dysfunction, disrupted lipid homeostasis, and systemic inflammation. Our results emphasize that HS diet–induced hypertensive injury is not solely a renal or CVD but also involves dynamic hepatic changes that may amplify cardiovascular risk through distinct molecular pathways and require further investigation.

The cardiac consequences of salt-induced hypertension have historically been attributed to chronic pressure overload and neurohumoral activation (37–39). However, accumulating evidence suggests that local metabolic and inflammatory remodeling contribute independently to cardiac hypertrophy and dysfunction (40–43). Our study reveals that the heart mounts an early proliferative stress response, which transitions into inflammatory and fibrotic remodeling, pointing to intrinsic molecular triggers rather than just mechanical load. This sequence mirrors earlier observations of hypertrophic cardiomyocyte signaling gradually shifting into maladaptive inflammation and tissue remodeling (44–46). Notably, our findings highlight IL-6/JAK/STAT3 as a central nonhemodynamic mediator in the heart’s response to hypertension (47). This is consistent with preclinical models where IL-6 deficiency reduces angiotensin II–induced cardiac fibrosis (48). Activation of JAK/STAT3 in this context is known to amplify cardiac inflammation and fibrosis even in the absence of sustained pressure overload (49). Together, these insights suggest that HS diet–induced hypertensive injury engages both mechanical and immune–metabolic pathways in the heart, with STAT3-dependent signaling emerging as a potential driver of maladaptive remodeling. Targeting this axis at the critical transition from proliferative stress to inflammatory remodeling may represent a promising strategy to prevent progression to heart failure in salt-induced hypertension.

Along with organ-specific responses, the identification of the shared proliferative program across organs is noteworthy, as most prior work has emphasized immune and fibrotic remodeling as central drivers of hypertensive kidney damage. Our data suggest that HS diet–induced hypertension initially elicits a coordinated proliferative response, which may reflect an adaptive attempt at tissue repair. Similar early activation of cell cycle networks has been observed in kidney injury (50, 51) and cardiac hypertrophy (52). However, when this response is inadequately controlled or sustained by long-term stress, it often precedes the transition to maladaptive remodeling (53, 54). We found that in progressive salt-induced hypertensive injury, a shared proliferative phase shifts to organ-specific immune and extracellular-matrix programs. Our longitudinal, multiorgan atlas traces this divergence toward organ-specific pathology and highlights its importance for future studies.

At the level of transcriptional regulation, beyond the identification of tissue-specific regulators, we identified BCL6 and RUNX1 as opposing master regulators, revealing a unifying mechanistic axis that integrates proliferative, inflammatory, and fibrotic responses across organs. BCL6 has been shown as a suppressor of NF-κB–dependent cytokine expression, with studies showing that its overexpression ameliorates renal, hepatic, and vascular inflammation (55–58). Conversely, RUNX1 has emerged as a critical driver of proinflammatory and profibrotic gene expression across multiple cell types, including macrophages, vascular smooth muscle cells, and tubular epithelial cells (59, 60). Our observation of consistent *Bcl6* downregulation and *Runx1* upregulation across organs suggests a coordinated transcriptional switch that may be exploited therapeutically in HS diet–induced hypertensive injury. Of note, RUNX1 inhibition has been shown to be beneficial for pulmonary arterial hypertension and myocardial infarction (61–63), although its role in hypertension and associated multiorgan damage remains unexplored.

Integrating transcriptomic signatures with human GWAS data allowed us to examine our findings through the lens of established genetic risk loci for hypertension and CKD. This approach not only strengthens the biological relevance of our experimental findings but also points to evolutionarily conserved pathways that may shape disease susceptibility. In this context, folate metabolism (*MTHFR*), steroidogenesis (*CYP17A1*), and extracellular matrix regulation (*COL4A1*, *COL6A3*) aligned with hypertension risk, while fatty acid oxidation (*ACADL*, *ACOX1*), solute transport (*SLC22A1* and *SLC22A2*), and tryptophan metabolism (*KYNU*, *KMO*) were linked to CKD risk. Among these, MTHFR is particularly notable, pointing to conserved one-carbon metabolism, which is not fully understood in salt-induced hypertension. MTHFR is a critical enzyme that regulates homocysteine–methionine balance and supplies methyl groups for DNA and histone methylation, thereby shaping epigenetic patterns that regulate vascular tone, renal sodium handling, and inflammatory signaling (64). Dysregulation of MTHFR has been associated with endothelial dysfunction and increased susceptibility to CVD (65, 66), yet its relevance to hypertension has not been systematically explored. Similarly, the kynurenine arm of tryptophan metabolism contributes to immunoregulation, redox balance, and NAD^+^ biosynthesis (67). Previously, we have shown the protective role of the kynurenine pathway against ischemic acute kidney injury in mice (68). However, in the Dahl SS rat model, and especially in the settings of hypertension and associated CKD, the role of the kynurenine pathway is not well characterized. These conserved mechanisms underscore the translational importance of our findings and position metabolic–immune cross-talk as a central driver of hypertension progression.

Organ damage in salt-induced hypertension often progresses despite intensive blood pressure control. High sodium itself promotes vascular dysfunction, oxidative stress, and fibrosis, including arterial stiffening via profibrotic mediators such as TGF-β, even in normotensive conditions (69–71). Therefore, observed effects in our study should be attributed to both salt loading and associated hypertension. Furthermore, whether observed changes in gene expression are reversible after salt withdrawal needs to be explored. Based on prior studies, early responses could be mainly salt-driven and might be reversed by early salt withdrawal (72–74). However, different pathways are expected to exhibit distinct degrees and time scales of reversibility, and these dynamics are likely to depend on the specific stage at which salt withdrawal occurs. These findings indicate that HS diet–induced hypertensive injury is not simply a secondary consequence of elevated blood pressure but reflects parallel molecular processes driven by both salt loading and hypertension that require better understanding and targeted therapeutic strategies.

Preclinical studies have demonstrated that interventions directed at hypertrophy, inflammation, or fibrosis can mitigate organ damage, yet these benefits are often highly context dependent and diminish when therapies are applied outside the optimal disease window (75–80). Our pharmacotranscriptomic analysis provides an explanation for this variability by suggesting that different classes of compounds may work better to rescue different stages of hypertensive injury. Our analysis suggests that drugs effective in one phase may lose efficacy if applied at another. This suggests that therapy for SS hypertension cannot be determined by blood pressure control alone but must also take into account the evolving molecular and functional state of the organs. Improved biomarkers that capture organ-specific trajectories are therefore needed to guide intervention timing and choice. Together, these insights argue for stage- and organ-tailored therapeutic strategies that move beyond conventional one-size-fits-all blood pressure management.

Despite its strengths, this study has limitations. We used mRNA-seq to achieve high coverage and statistical power to generate a detailed transcriptomic view of HS diet–induced hypertensive injury. However, this approach cannot resolve cell type–specific transcriptional heterogeneity. We prioritized depth and sensitivity, which remain limited with current single-cell technologies. Recently, recognizing the importance of cellular resolution, research efforts have initiated single-cell mapping of hypertension (81). More studies using single-cell and spatial transcriptomics will be essential to define both cell type– and time-specific contributions with greater precision. Additionally, although the Dahl SS rat is a well-established model of human salt-induced hypertension, which is also supported by our GWAS analysis, species differences should be considered when translating these findings to humans.

In summary, our study presents a transcriptomic atlas of salt-induced hypertensive injury progression across the kidney, liver, and heart, which can be leveraged to explore diverse mechanistic pathways.

## Methods

### Sex as a biological variable.

Male Dahl SS rats were used in this study. Sex was not directly evaluated as a biological variable. Because HS diet–induced hypertensive injury has been observed in both male and female Dahl SS rats, we expect that many of the core pathophysiologic pathways identified here will be relevant to female rats. However, sex-dependent differences in the magnitude and temporal progression of these responses are possible.

### Animals.

Male Dahl SS rats (SS/JrHsdMcwi) were housed in the AAALAC-accredited animal facility at the University of South Florida under specific pathogen-free conditions, maintained on a 12-hour light/dark cycle in temperature- and humidity-controlled rooms, with ad libitum access to food and water. Experimental animals were housed in the same cage-holding rack within the same room under identical environmental conditions to minimize location effects. Treatments and measurements were performed in randomized order to reduce potential bias.

Dahl SS rats were maintained on a NS diet (0.4% NaCl, D113755, Dyets Inc.). To induce hypertension, 9- to 11-week-old animals were switched to a HS diet (4% NaCl, D113756, Dyets Inc) for 7, 14, 21, or 35 days. Animals on NS diet were used as control groups. For all experiments, animals were randomly assigned to control and treatment groups. One day before the final day of selected time points (day 7, 14, 21, and 35), rats were individually housed in metabolic cages for 24-hour urine collection. At each time point, animals were euthanized under deep isoflurane/O_2_ anesthesia and prior to renal perfusion, heparinized blood was obtained from the descending aorta and centrifuged at 5,000*g* for 5 minutes to isolate plasma. Tissue samples were collected and snap-frozen in liquid nitrogen or fixed in formalin for transcriptomic and histological assessments. Blood and urine samples were used for biochemical profiling. No inclusion or exclusion criteria were applied. All animals were retained in the study unless removal was recommended by animal care staff for welfare reasons.

### Blood pressure measurement.

We monitored blood pressure for 21 days on a different cohort of Dahl SS rats using the DSI telemetry system to confirm the blood pressure elevation with HS diet and establish the hypertensive phenotype as described previously (19, 82).

### RNA-seq and analysis.

Total RNA was isolated from snap-frozen tissues and submitted for library preparation and paired-end Illumina sequencing. High-quality RNA samples were used for sequencing, and clean reads were aligned to the *Rattus norvegicus* reference genome (Ensembl Rnor_6.0) using HISAT2. Gene-level counts were generated with featureCounts, and differential expression analysis was performed using DESeq2, with genes meeting |log_2_ fold change| ≥ 0.585 and *P*_adj_ < 0.05 considered differentially expressed. Downstream analyses included dimensionality reduction, correlation analysis, pathway enrichment, TF analysis, PPI network analysis, transcriptomic-GWAS integration, and drug perturbation analysis. Histopathology, and serum and urine biochemical analyses were also performed. The sample size (*n* = 6 per group) was chosen based on our previous studies, which demonstrated that this number of biological replicates is sufficient to ensure reliable RNA sequencing results and capture biologically meaningful differences (83). Detailed experimental protocols and computational workflows are provided in the Supplemental Methods.

### Statistics.

Statistical analyses were performed using GraphPad Prism (v10.5) and R (v4.4.0). For RNA-seq data, differential gene expression was assessed using DESeq2. Genes with an |log_2_ fold change| ≥ 0.585, equivalent to a fold change ≥ 1.5, *P*_adj_ < 0.05 were considered significantly differentially expressed. Statistical significance was assessed using 1-way or 2-way ANOVA followed by Šídák’s multiple comparisons test for multiple groups. Data are presented as mean ± SEM, and *P* < 0.05 was considered statistically significant.

### Study approval.

All animal procedures were conducted in accordance with the Guide for the Care and Use of Laboratory Animals (National Academies Press, 2011) and were approved by the Institutional Animal Care and Use Committee (IACUC) at the University of South Florida.

### Data availability.

The RNA-seq data can be found at Gene Expression Omnibus (GEO) under accession number GSE305010. Furthermore, data can be explored in an online data resource at *STAR LAB-Hypertension Atlas* (https://starlabusf.com/). Supporting data values associated with the figures are provided in the Supporting Data Values file. Additional data and supporting materials are available from the corresponding authors upon reasonable request.

## Author contributions

RT and AS conceptualized and designed the study. RT, VL, and ML performed the experiments and collected samples. RT, OK, LVD, ML, BX, RB, SD, DVI, and OP analyzed the data. RT and AS interpreted the data. RT wrote the manuscript and finalized it for publication. All authors reviewed and approved the final version of the article.

## Conflict of interest

The authors have declared that no conflict of interest exists.

## Funding support

This work is the result of U.S. Department of Veterans Affairs grants and National Institutes of Health (NIH) funding, in whole or in part, and is subject to the NIH Public Access Policy. Through acceptance of this federal funding, the NIH has been given a right to make the work publicly available in PubMed Central. The contents do not represent the views of the Department of Veterans Affairs or the United States Government.

- U.S. Department of Veterans Affairs grant IK6 RD001204 (to AS).
- U.S. Department of Veterans Affairs grant I01BX004024 (to AS).
- NIH grants R01 DK135644 (to AS).
- NIH grants R01 DK129227 (to AS and OP).
- NIH grants R01 HL148114 (to DVI).
- American Heart Association 26RIRA1622963 (to RT).
- Vascular Inflammation and Injury Training Program T32 HL160529 (to RB).
- The Dialysis Clinic Inc. Paul Teschan Research Fund (to OP.)
- USF Hypertension and Kidney Research Center Early Investigator Awards (to RT, OK, and LVD) and Multi-PI Award (AS).
- NIH grants K99 DK143296 (to BX).
- American Heart Association 25POST1375066 (to LVD).

## Supplementary Material

Supplemental data

Supplemental data set 1

Supplemental data set 10

Supplemental data set 11

Supplemental data set 12

Supplemental data set 2

Supplemental data set 3

Supplemental data set 4

Supplemental data set 5

Supplemental data set 6

Supplemental data set 7

Supplemental data set 8

Supplemental data set 9

Supporting data values

## Version 1 04/23/2026

In-Press Preview

## Version 2 06/22/2026

Electronic publication

## References

[1] GBD 2017 Causes of Death Collaborators (2018). Global, regional, and national age-sex-specific mortality for 282 causes of death in 195 countries and territories, 1980–2017: a systematic analysis for the Global Burden of Disease Study 2017. Lancet.

[2] GBD 2017 Risk Factor Collaborators (2018). Global, regional, and national comparative risk assessment of 84 behavioural, environmental and occupational, and metabolic risks or clusters of risks for 195 countries and territories, 1990–2017: a systematic analysis for the Global Burden of Disease Study 2017. Lancet.

[3] Mills KT (2020). The global epidemiology of hypertension. Nat Rev Nephrol.

[4] Mills KT (2016). Global disparities of hypertension prevalence and control: a systematic analysis of population-based studies from 90 countries. Circulation.

[5] Bailey MA, Dhaun N (2024). Salt sensitivity: causes, consequences, and recent advances. Hypertension.

[6] Nishimoto M (2024). Salt-sensitive hypertension and the kidney. Hypertension.

[7] Flores J Salt sensitivity of blood pressure and the role of the immune system in hypertension. Cardiol Rev.

[8] Laster M (2018). Kidney disease among African Americans: a population perspective. Am J Kidney Dis.

[9] Vogt L (2023). Novel mechanisms of salt-sensitive hypertension. Kidney Int.

[10] Majid DS (2015). Salt-sensitive hypertension: perspectives on intrarenal mechanisms. Curr Hypertens Rev.

[11] Peters RM, Flack JM (2000). Salt sensitivity and hypertension in African Americans: implications for cardiovascular nurses. Prog Cardiovasc Nurs.

[12] Ou-Yang YN (2022). Revealing the pathogenesis of salt-sensitive hypertension in Dahl salt-sensitive rats through integrated multi-omics analysis. Metabolites.

[13] Gao P (2022). Salt-induced hepatic inflammatory memory contributes to cardiovascular damage through epigenetic modulation of SIRT3. Circulation.

[14] Dahl LK (1962). Role of genetic factors in susceptibility to experimental hypertension due to chronic excess salt ingestion. Nature.

[15] Rapp JP (1982). Dahl salt-susceptible and salt-resistant rats. A review. Hypertension.

[16] Rapp JP (2000). Genetic analysis of inherited hypertension in the rat. Physiol Rev.

[17] (2006). The genetic dissection of essential hypertension. Nat Rev Genet.

[18] Abais-Battad JM (2019). Dietary effects on Dahl salt-sensitive hypertension, renal damage, and the T lymphocyte transcriptome. Hypertension.

[19] Rinschen MM (2022). Accelerated lysine metabolism conveys kidney protection in salt-sensitive hypertension. Nat Commun.

[20] Rinschen MM (2019). Metabolic rewiring of the hypertensive kidney. Sci Signal.

[21] De Miguel C (2010). T lymphocytes mediate hypertension and kidney damage in Dahl salt-sensitive rats. Am J Physiol Regul Integr Comp Physiol.

[22] Fehrenbach DJ (2019). Salt-sensitive increase in macrophages in the kidneys of Dahl SS rats. Am J Physiol Renal Physiol.

[23] Szklarczyk D (2023). The STRING database in 2023: protein-protein association networks and functional enrichment analyses for any sequenced genome of interest. Nucleic Acids Res.

[24] Garcia-Alonso L (2019). Benchmark and integration of resources for the estimation of human transcription factor activities. Genome Res.

[25] Lachmann A (2010). ChEA: transcription factor regulation inferred from integrating genome-wide ChIP-X experiments. Bioinformatics.

[26] Zheng Q (2025). Bidirectional histone monoaminylation dynamics regulate neural rhythmicity. Nature.

[27] Blondel VD (2008). Fast unfolding of communities in large networks. J Stat Mech.

[28] Keenan AB (2018). The library of integrated network-based cellular signatures NIH program: system-level cataloging of human cells response to perturbations. Cell Syst.

[29] Subramanian A (2017). A next generation connectivity map: L1000 platform and the first 1,000,000 Profiles. Cell.

[30] (2024). Renal medulla in hypertension. Hypertension.

[31] Zhao YC (2020). Nonalcoholic fatty liver disease: an emerging driver of hypertension. Hypertension.

[32] Motamed N (2017). Non-alcoholic fatty liver disease (NAFLD) and 10-year risk of cardiovascular diseases. Clin Res Hepatol Gastroenterol.

[33] Huh JH (2015). High dietary sodium intake assessed by estimated 24-h urinary sodium excretion is associated with NAFLD and hepatic fibrosis. PLoS One.

[34] Choi Y (2016). Dietary sodium and potassium intake in relation to non-alcoholic fatty liver disease. Br J Nutr.

[35] Wu S (2016). Association of non-alcoholic fatty liver disease with major adverse cardiovascular events: a systematic review and meta-analysis. Sci Rep.

[36] Chen H (2025). Adding salt to foods increases the risk of metabolic dysfunction-associated steatotic liver disease. Commun Med (Lond).

[37] Guyton AC (1991). Blood pressure control--special role of the kidneys and body fluids. Science.

[38] Susic D (2009). Angiotensin blockade prevents salt-induced injury of the renal circulation in spontaneously hypertensive rats. Am J Nephrol.

[39] Fujita T (2014). Mechanism of salt-sensitive hypertension: focus on adrenal and sympathetic nervous systems. J Am Soc Nephrol.

[40] Savoia C (2011). Vascular inflammation and endothelial dysfunction in experimental hypertension. Int J Hypertens.

[41] Touyz RM (2000). Oxidative stress and vascular damage in hypertension. Curr Hypertens Rep.

[42] Ritterhoff J (2020). Metabolic remodeling promotes cardiac hypertrophy by directing glucose to aspartate biosynthesis. Circ Res.

[43] Li J (2019). Metabolic changes in spontaneously hypertensive rat hearts precede cardiac dysfunction and left ventricular hypertrophy. J Am Heart Assoc.

[44] Burchfield JS (2013). Pathological ventricular remodeling: mechanisms: part 1 of 2. Circulation.

[45] Hill JA, Olson EN (2008). Cardiac plasticity. N Engl J Med.

[46] van Berlo JH (2013). Signaling effectors underlying pathologic growth and remodeling of the heart. J Clin Invest.

[47] Hilfiker-Kleiner D (2006). Molecular mechanisms in heart failure: focus on cardiac hypertrophy, inflammation, angiogenesis, and apoptosis. J Am Coll Cardiol.

[48] Ma F (2012). Macrophage-stimulated cardiac fibroblast production of IL-6 is essential for TGF β/Smad activation and cardiac fibrosis induced by angiotensin II. PLoS One.

[49] Chen F (2017). Interleukin-6 deficiency attenuates angiotensin II-induced cardiac pathogenesis with increased myocyte hypertrophy. Biochem Biophys Res Commun.

[50] Galichon P (2024). Energy depletion by cell proliferation sensitizes the kidney epithelial cells to injury. Am J Physiol Renal Physiol.

[51] DiRocco DP (2014). CDK4/6 inhibition induces epithelial cell cycle arrest and ameliorates acute kidney injury. Am J Physiol Renal Physiol.

[52] Ahuja P (2007). Cardiac myocyte cell cycle control in development, disease, and regeneration. Physiol Rev.

[53] Schiattarella GG, Hill JA (2015). Inhibition of hypertrophy is a good therapeutic strategy in ventricular pressure overload. Circulation.

[54] Lee K (2021). Epithelial proliferation and cell cycle dysregulation in kidney injury and disease. Kidney Int.

[55] Chen D (2017). BCL6 attenuates renal inflammation via negative regulation of NLRP3 transcription. Cell Death Dis.

[56] Gu Y (2025). BCL6 alleviates hepatic ischemia/reperfusion injury via recruiting SIRT1 to repress the NF-κB/NLRP3 pathway. Transplantation.

[57] Sommars MA (2019). Dynamic repression by BCL6 controls the genome-wide liver response to fasting and steatosis. Elife.

[58] Liongue C (2024). B cell lymphoma 6 (BCL6): a conserved regulator of immunity and beyond. Int J Mol Sci.

[59] Zhou T (2018). Runt-related transcription factor 1 (RUNX1) promotes TGF-β-induced renal tubular epithelial-to-mesenchymal transition (EMT) and renal fibrosis through the PI3K subunit p110δ. EBioMedicine.

[60] Dubey S (2022). Inhibition of RUNX1 blocks the differentiation of lung fibroblasts to myofibroblasts. J Cell Physiol.

[61] Riddell A (2020). RUNX1: an emerging therapeutic target for cardiovascular disease. Cardiovasc Res.

[62] Martin TP (2023). Ribonucleicacid interference or small molecule inhibition of Runx1 in the border zone prevents cardiac contractile dysfunction following myocardial infarction. Cardiovasc Res.

[63] Jeong EM (2022). Targeting RUNX1 as a novel treatment modality for pulmonary arterial hypertension. Cardiovasc Res.

[64] Crider KS (2012). Folate and DNA methylation: a review of molecular mechanisms and the evidence for folate’s role. Adv Nutr.

[65] Klerk M (2002). MTHFR 677C-->T polymorphism and risk of coronary heart disease: a meta-analysis. JAMA.

[66] Raghubeer S, Matsha TE (2021). Methylenetetrahydrofolate (MTHFR), the one-carbon cycle, and cardiovascular risks. Nutrients.

[67] Xue C (2023). Tryptophan metabolism in health and disease. Cell Metab.

[68] Torosyan R (2021). Hypoxic preconditioning protects against ischemic kidney injury through the IDO1/kynurenine pathway. Cell Rep.

[69] Grigorova YN (2018). Dietary sodium restriction reduces arterial stiffness, vascular TGF-β-dependent fibrosis and marinobufagenin in young normotensive rats. Int J Mol Sci.

[70] DuPont JJ (2013). High dietary sodium intake impairs endothelium-dependent dilation in healthy salt-resistant humans. J Hypertens.

[71] Matthews EL (2015). High dietary sodium reduces brachial artery flow-mediated dilation in humans with salt-sensitive and salt-resistant blood pressure. J Appl Physiol (1985).

[72] McLoone VI (2009). A multi-component model of the dynamics of salt-induced hypertension in Dahl-S rats. BMC Physiol.

[73] Van Vliet BN (2006). Distinct rapid and slow phases of salt-induced hypertension in Dahl salt-sensitive rats. J Hypertens.

[74] Arkhipov SN (2024). Dissociation of hypertension and renal damage after cessation of high-salt diet in Dahl rats. Hypertension.

[75] McMullen JR (2004). Inhibition of mTOR signaling with rapamycin regresses established cardiac hypertrophy induced by pressure overload. Circulation.

[76] Hilfiker-Kleiner D (2010). Continuous glycoprotein-130-mediated signal transducer and activator of transcription-3 activation promotes inflammation, left ventricular rupture, and adverse outcome in subacute myocardial infarction. Circulation.

[77] Guo B (2024). Targeting the JAK2/STAT3 signaling pathway with natural plants and phytochemical ingredients: a novel therapeutic method for combatting cardiovascular diseases. Biomed Pharmacother.

[78] Di X (2025). Targeting fibrosis: from molecular mechanisms to advanced therapies. Adv Sci (Weinh).

[79] Bueno OF (2000). The MEK1-ERK1/2 signaling pathway promotes compensated cardiac hypertrophy in transgenic mice. EMBO J.

[80] Mohammed KAK (2024). MEK inhibitors: a promising targeted therapy for cardiovascular disease. Front Cardiovasc Med.

[82] Palygin O (2017). Essential role of Kir5.1 channels in renal salt handling and blood pressure control. JCI Insight.

[83] Khedr S (2024). Role of cGAS/STING pathway in aging and sexual dimorphism in diabetic kidney disease. JCI Insight.

